# Plant biosecurity and One Health: government and industry roles as risk creators and mitigators

**DOI:** 10.1186/s42522-025-00150-y

**Published:** 2025-04-25

**Authors:** John I. Alawneh, Mohammad Mahmudul Hassan, James Camac, Lois Ransom, James Planck, Susan C. Porchun, Michael Reid, Rachel Chay

**Affiliations:** 1 Plant Biosecurity and Product Integrity, Biosecurity Queensland, Department of Primary Industries, Brisbane, 4000 Australia; 2 Epidemiology and Data Integrity (EDIT), Plant Biosecurity and Product Integrity, Biosecurity Queensland, Department of Primary Industries, Brisbane, 4000 Australia; 3 Queensland Alliance for One Health Sciences, School of Veterinary Science, The University of Queensland, Gatton, QLD 4343 Australia; 4 Centre of Excellence for Biosecurity Risk Analysis (CEBRA), School of BioSciences, The University of Melbourne, Victoria, Australia; 5 Lois Ransom PSM, LMR Consulting, Canberra, ACT 2904 Australia; 6 Biosecurity Queensland, Department of Primary Industries, Brisbane, 4000 Australia

**Keywords:** Plant biosecurity, One health, Governance, Industry, Surveillance

## Abstract

The One Health concept highlights the interconnectedness of human, animal, and environmental health and places significant importance on plant biosecurity. This is due to the profound impact of plant biosecurity on food safety and security for animals and people, biodiversity, and the economy. This narrative review examines the roles of government and industry as risk creators and mitigators in plant biosecurity within a One Health framework, focusing on how their collaboration can strengthen surveillance, enhance regulatory policies, and mitigate the spread of plant pests and diseases. Plant biosecurity, which encompasses the measures to safeguard plant biosecurity and life in the same way that animal biosecurity safeguards animal and human health and life, is a critical component of One Health. Measures include a range of policies, regulations, strategies and activities to protect plants from exotic and established pests and diseases. Government, industry, and community actions are critical elements of plant biosecurity. These include pest surveillance and the establishment and maintenance of pest-free areas. Government agencies and industry professionals play a central and pivotal role in shaping plant biosecurity by implementing policies and regulations and developing innovative strategies. These actions can have a dual effect on plant biosecurity: they can either mitigate risks by preventing the introduction and spread of pests or create risks if regulations are inadequate or poorly enforced. The success of plant biosecurity efforts depends on how well government policies align with One Health principles, which require a careful balance between economic, environmental, social and health-related technical/scientific considerations. Pest surveillance, a foundational element of plant biosecurity, provides the tools for early detection and rapid response to pest outbreaks, essential for protecting plant biosecurity. Surveillance programs enable continuous monitoring of pest populations and the detection of emerging threats, which is critical for maintaining pest-free areas. The benefits of pest surveillance are numerous and extend beyond plant biosecurity, contributing to broader One Health objectives by reducing the risk of zoonotic diseases and preserving the ecological integrity of ecosystems. It underpins important economic and trade objectives by projecting confidence in the safety and health of Australia’s agricultural products to international trading partners. Strategies to achieve and maintain pest-free areas include stringent quarantine measures, continuous surveillance, and effective rapid response protocols. The interconnectedness of plant biosecurity with One Health is evident in these efforts, as maintaining pest-free areas supports ecosystem health, minimises the need for chemical interventions and consequent pressure on antimicrobial resistance, and promotes sustainable agricultural practices. Government actions, pest surveillance, and the maintenance of pest-free regions are essential components of a robust plant biosecurity strategy. By aligning these measures with One Health principles, it is possible to protect plant biosecurity, enhance environmental sustainability, and contribute to global health outcomes. This holistic approach highlights the importance of cross-sector collaboration and the need for solid biosecurity frameworks to safeguard plant biosecurity in an increasingly interconnected world.

## Highlights

Plant biosecurity is vital for biodiversity, and the economy and is a key element of One Health.

Effective government policies and regulations are essential for preventing the spread of plant pests and diseases, aligning with One Health principles and other One Health disciplines.

Continuous and effective pest surveillance ensures early detection and rapid response to outbreaks, supporting both plant biosecurity and broader ecosystem services.

Other biosecurity measures, including control, containment, quarantine and eradication, are essential for maintaining pest-free areas, promoting sustainable agriculture, and strengthening global health outcomes.

## The One Health concept and plant biosecurity

The One Health concept, which integrates human, animal, and environmental health, highlights the interconnectedness of all living organisms, promoting a holistic approach to global health challenges [1]. Plant biosecurity and biosecurity are fundamental within this framework. Healthy plants are essential for ecological balance, food security, and human well-being, making plant biosecurity, the measures to safeguard and enhance plant health and life, a critical component of the One Health paradigm [2]. This short document explores how government and industry actions and policies can impact plant biosecurity and, consequently, the broader One Health framework, which integrates the health of humans, animals, and the environment [3]. Effective plant biosecurity measures, including pest surveillance and quarantine, are necessary to protect plant biosecurity and, by extension, human and animal health [4], plant biosecurity and food security [5]. The spread of plant pathogens can also contribute to zoonotic disease risks, demonstrating the complex interdependencies within One Health [6, 7]. Ensuring plant biosecurity through sustainable practices helps maintain the ecosystem services vital for life on Earth [8]. Cross-sector collaboration is essential to addressing the challenges posed by plant biosecurity within the One Health framework [9].

Plants are at the core of terrestrial life and produce oxygen, sequester carbon, and provide food, fibre, and medicine. Plant biosecurity affects the entire food web, from soil microorganisms to apex predators, including humans. Healthy plants support biodiversity by providing habitats and food sources for various species, thus maintaining ecosystem stability [10]. The Food and Agriculture Organization (FAO) estimates that plant diseases and pests cause up to 40% of global food crop losses annually, significantly impacting food security and agricultural economies [11]. The Irish Potato Famine in the 1840s, caused by *Phytophthora infestans*, exemplifies the catastrophic impact plant diseases can have on societies [12]. Ensuring plant biosecurity is not just an agricultural priority but a global imperative. Thus, integrating plant biosecurity into the One Health approach is vital for achieving global health and environmental sustainability. The One Health concept highlights the interconnectedness of human, animal, and environmental health, emphasising the importance of plant biosecurity within this framework. In Australia, “One Biosecurity” emerged from a 2008 review of quarantine and biosecurity measures, advocating for stronger federal and state partnerships to combat agricultural pests and diseases [13]. Safeguarding plant biosecurity is crucial for preventing the spread of pests and diseases, which can significantly impact food security, biodiversity, and ecosystem health. Integrating plant biosecurity into the One Health approach is increasingly recognised as essential for managing biosecurity risks and promoting sustainable agricultural practices in the country [14]. The effects of plant biosecurity practices on human health are often overlooked in One Health discussions. Actively recognising plant biosecurity within the One Health framework emphasises the balance between food security and ecological sustainability, promoting comprehensive approaches that address agricultural needs while enhancing ecosystem, animal, and human health [15]. Animal and human health often integrate strategies like vaccination and hygiene measures to reduce disease transmission [16, 17]. In contrast, plant health leans on surveillance, pest management, and biosecurity practices like quarantines and resistant crop varieties [18]. Clear areas of overlap are chemical and non-chemical control of pests, disease agents and vectors and managing risks of antimicrobial resistance. Combining these perspectives in biosecurity strengthens the system by preventing cross-sector threats, such as zoonoses that impact animals and plants and ensuring a more resilient food system [19]. Effective biosecurity measures require cooperation between sectors, utilising shared strategies and technologies to safeguard ecosystems, economies, and public health [20, 21]. Plant biosecurity is crucial as it supports food security, directly impacting human and animal health through the availability of safe, nutritious crops and feed [22, 23]. Healthy plants prevent the spread of pests and diseases that can devastate ecosystems, leading to economic loss and scarcity of resources, weakening animal populations and heightening the risks of zoonotic diseases [24]. Furthermore, robust plant biosecurity helps maintain environmental balance, reducing the need for chemical interventions that can harm biodiversity and contribute to antimicrobial resistance [25, 26].

## Plant biosecurity: interconnectedness with One Health

The health of plants is intrinsically connected to the One Health concept, emphasising the interdependence of human, animal, and environmental health. Actions by government agencies and industry significantly impact this interconnectedness. Healthy plant ecosystems regulate diseases, reducing the risk of zoonotic diseases that can spread from animals to humans [27]. Invasive species and plant pathogens can disrupt ecosystems, leading to biodiversity loss and altering habitats in ways that increase disease transmission [28, 29]. Pest outbreaks that reduce crop yields can lead to food shortages and malnutrition, affecting human health. Some plant pathogens generate mycotoxins, such as aflatoxin, that adversely impact animal and human health. Additionally, some plant pests can pose human health risks, such as *Claviceps purpurea* (ergot), which produces toxic alkaloids [30], and *Hylesia nigricans* (burning moth), whose hairs cause dermatitis in humans [31]. Moreover, the brown marmorated stink bug (*Halyomorpha halys*) can affect fruit crops, impacting food availability [32]. Plant biosecurity impacts animal health by influencing the availability and quality of forage and habitat in natural and production systems. Invasive plant species can alter ecosystems, reducing the availability of native plants that animals rely on for food and shelter. This can decrease biodiversity and ecosystem resilience [33, 34]. Plant biosecurity is deeply interconnected with the One Health framework as plants form the foundation of global food systems [22]. In recent years, vegetables such as romaine lettuce have increasingly been linked to food-borne illnesses like *E. coli* infections in the USA [35, 36], with many plant-based foods now associated with previously unexpected outbreaks. Enteric zoonotic pathogens, typically found in animals and transmitted to plants through manure-contaminated irrigation and washing water, are often responsible for these outbreaks. Adopting a One Health approach, which integrates animal, human, and environmental health, offers practical interventions to reduce pathogen transmission while safeguarding plant and animal health [37].

Plants are critical in maintaining the integrity of ecosystem processes, such as carbon sequestration, water regulation, and maintenance of soil health. Invasive pests and diseases can disrupt these processes, leading to environmental degradation. Effective plant biosecurity measures help preserve ecosystem health and biodiversity, supporting overall environmental stability [38]. The globalisation of trade and climate change have intensified the scope and frequency of international movements of pests and pathogens, making plant biosecurity crucial for preventing the spread of harmful organisms. Integrated approaches that combine plant biosecurity surveillance with broader One Health monitoring systems can enhance the early detection and management of emerging threats [39]. Collaboration across disciplines, including agriculture, ecology, and public health, is necessary to address the complex challenges of plant pests and diseases. Protecting plant biosecurity safeguards the well-being of humans, animals, and ecosystems, contributing to sustainable development and global health. The interconnectedness of plant biosecurity and One Health highlights the need for holistic strategies to manage the health of our planet.

### The role of plant biosecurity in the One Health approach

**Fig. 1:**
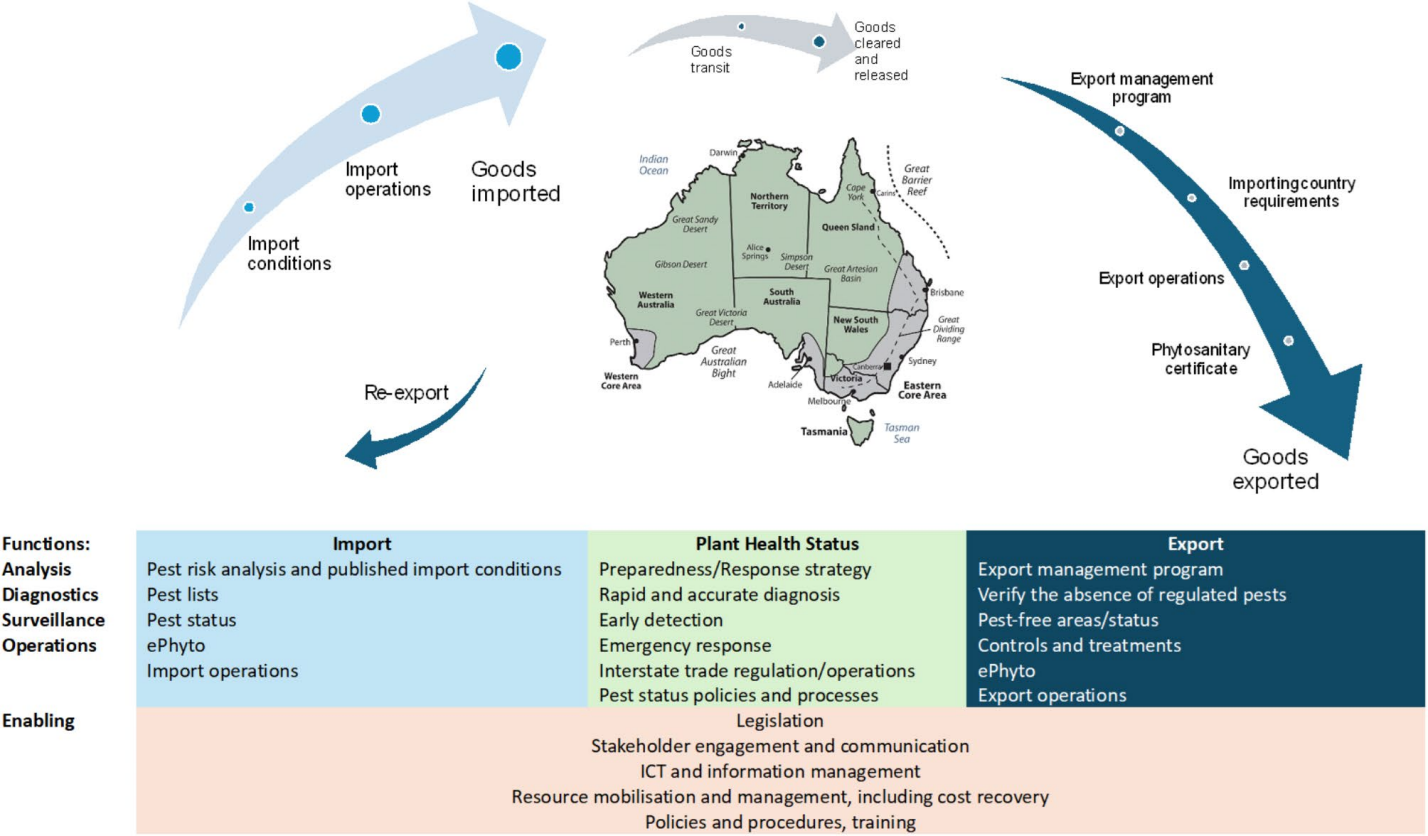
Graphic illustrating the Australian plant biosecurity landscape and phytosanitary trading system, based on the IPPC framework, showing the interconnected elements that safeguard plant resources and define the roles and responsibilities of government, farmers, and communities in promoting shared biosecurity responsibility (Ransom, L. Pers. Comm.)

Plant biosecurity is often overlooked in One Health discussions yet integrating it can enhance ecological health by balancing food security with planetary boundaries. A more inclusive approach to plant protection interventions can generate co-benefits for ecosystems, animals, and humans. Strengthening the capacity of regulatory bodies to conduct cost-benefit analyses is crucial for evaluating trade-offs in One Health interventions across diverse contexts [40]. Plant biosecurity encompasses all measures to protect plants biosecurity from pests, diseases, and invasive species. Effective biosecurity practices are essential for preventing the introduction and spread of harmful organisms. These practices include quarantine regulations, import restrictions, and the deployment of early detection and rapid response systems. The International Plant Protection Convention (IPPC) and World Trade Organisation (WTO) Agreement on the Application of Sanitary and Phytosanitary Measures set and enforce, respectively, global standards for these phytosanitary measures, helping countries collaborate to protect plant health [41, 42]. Plant biosecurity in Australia is crucial for safeguarding the nation’s agricultural productivity, biodiversity, and natural ecosystems from invasive pests and diseases (Fig. 1). It involves comprehensive measures, including quarantine protocols, surveillance, and rapid response strategies, to prevent the introduction and spread of biosecurity threats. Effective plant biosecurity is vital for ensuring food security, protecting the economy, and maintaining Australia’s unique environmental heritage [43].

A country’s plant health status, determined by the presence or absence of pests, sets the phytosanitary conditions for trade and identifies priority pests for biosecurity efforts. The national plant health system manages risks to the economy, environment, and community, as outlined by the Council of Australian Governments in 2019 [44] and supported by the Australian Intergovernmental Agreement on Biosecurity (IGAB). The IGAB outlines key functions for biosecurity (Fig. 1), covering all hazards and supporting One Health initiatives. In Australia and New Zealand, the system concept highlights the interconnected elements needed to safeguard plant resources and facilitate trade while defining the roles and responsibilities of governments, farmers, and communities in a shared biosecurity approach. The “biosecurity continuum”, introduced by the Australian Quarantine Review Secretariat and Nairn [45] and adopted in the 2008 National Fruit Fly Strategy, emphasises the need for an uninterrupted biosecurity system across offshore, border, and onshore levels.

### Key components of plant biosecurity

#### Pest surveillance and monitoring

Regular monitoring and surveillance programs are essential for the early detection of pest outbreaks. Advanced technologies, such as remote sensing and molecular diagnostics, enhance the ability to efficiently identify and track pest populations. For example, drones and satellite imagery can help monitor large agricultural areas for signs of pest activity [46]. Moreover, monitoring pests in Australia, particularly through environmental DNA (eDNA) methods, can enhance biosecurity efforts by detecting invasive species that threaten biodiversity and agriculture. Targeted eDNA surveillance for high-risk groups such as insects, weeds, and marine biofouling organisms is essential to prevent the introduction and spread of non-native species in vulnerable ecosystems [47–49]. This involves monitoring and early detection of plant pests and pathogens to prevent their establishment and spread. Effective surveillance programs are critical for identifying emerging threats and enabling rapid responses [4]. Pest surveillance and monitoring in Australia are essential for protecting the country’s agricultural industries and natural ecosystems from invasive species. Programs such as the National Plant Health Surveillance Program (NPHSP), and National Bee Pest Surveillance Program (NBPSP) focus on early detection and control of pests like fruit flies (*Bactrocera dorsalis*) and Asian honey bees (*Apis cerana*), reducing the impact on crop production and biodiversity [50]. Recently, particularly for the Varroa mite (*Varroa destructor*), these programs have been critical to protecting the nation’s honeybee population and pollination services. The National Varroa Mite Eradication Program focuses on early detection through sentinel hives and regular surveillance of bee populations near ports to prevent the spread of this harmful pest [51, 52]. The 2022 Varroa mite detection in New South Wales has led to increased biosecurity measures and eradication efforts to contain the spread and mitigate the impact on agriculture [53, 54].

#### Rapid response

When a pest or disease is detected, a rapid response is critical to contain and eradicate the threat. This involves coordinated efforts between government agencies, researchers, and farmers. The successful eradication of the Mediterranean fruit fly (*Ceratitis capitata*) in California in the 1970s through sterile insect techniques illustrates the effectiveness of well-coordinated response strategies [55]. Preparedness for pest and disease outbreaks involves planning for rapid containment and eradication efforts. Effective emergency response can minimise the impact of biosecurity breaches [4]. Rapid biosecurity response is vital to mitigate the risks of invasive species such as the fire ant (*Solenopsis invicta*) in Australia. For example, the National Fire Ant Eradication Program (NFAEP) is a coordinated effort to detect and eliminate fire ant infestations through active surveillance, baiting, and destruction of nests [56]. The detection of fire ants in Queensland triggered immediate containment measures, including quarantines and treatment zones, to prevent their spread and protect agriculture and human health [57].

#### Quarantine and border controls

The most effective way to protect plant biosecurity is to prevent pests and diseases from entering new areas. This involves stringent border controls, sanitary and phytosanitary measures, including quarantine and treatment, and public awareness campaigns to educate about the risks of moving plants and plant products. Measures may be needed to prevent the introduction and spread of pests and diseases across borders. Quarantine regulations restrict the movement of plants and plant products that may harbour harmful organisms [58]. Government policies and international agreements play a vital role in enforcing plant biosecurity. Regulatory frameworks establish the legal basis for quarantine measures, pest management, and global trade standards [59]. Quarantine and border controls in Australia are pivotal in preventing the introduction and spread of harmful pests and diseases that could threaten the country’s agricultural and natural environments. These measures include rigorous inspection protocols, treatment requirements, and the management of import permits to ensure compliance with biosecurity standards [60]. Advanced surveillance systems and collaboration with international partners mitigate biosecurity risks, supporting effective quarantine and border controls in Australia [61].

#### Risk assessment and management

Assessing the risks associated with plant pests and diseases is essential for prioritising resources and implementing targeted biosecurity measures. Risk management strategies include developing contingency plans and response protocols [62]. After a pest or disease has crossed the border, post-border management involves containment, eradication, and long-term management to mitigate its impact [63, 64]. Risk assessment and management of plant biosecurity in Australia are key to safeguarding the country’s agriculture and natural ecosystems. The Australian Government uses a science-based risk analysis process, underpinned by the Biosecurity Act 2015, to assess threats from pests and diseases, such as citrus canker and myrtle rust [65]. These assessments guide the development of risk management strategies, including import conditions, surveillance programs, and emergency response plans. Effective risk management involves collaboration between government, industry, and research organisations to minimise the likelihood of pest introductions and ensure rapid action in case of outbreaks [66].

#### Integrated pest management (IPM)

IPM is an eco-friendly approach that combines biological, cultural, physical, and chemical tools to manage pests. By emphasising sustainable and scientifically sound practices, IPM reduces reliance on chemical pesticides, thus protecting the environment and human health [67]. IPM in Australia involves a holistic approach to managing pest populations by combining biological, cultural, physical, and chemical control methods. This strategy aims to minimise the use of chemical pesticides and reduce their environmental impact while effectively managing pest threats to agriculture and natural ecosystems [68]. Successful IPM implementation in Australia relies on ongoing research, monitoring, and collaboration between farmers, researchers, and industry stakeholders [69]. IPM strategies are employed to reduce the risk of pest outbreaks, such as those from Queensland fruit fly and silverleaf whitefly, while minimising environmental impacts [70, 71]. Australian government and industry initiatives promoting sustainable farming practices and using advanced technologies for monitoring and early intervention support the adoption of IPM in plant biosecurity [72, 73].

#### Pest surveillance: a pillar of plant biosecurity

Pest surveillance systematically collects, analyses, and interprets data on pest populations. A proactive approach enables early detection and rapid response to pest outbreaks. Effective surveillance is essential for establishing, maintaining and verifying pest-free areas and supporting the global trade of agricultural products.

### Benefits of pest surveillance

#### Early detection and control

Early detection allows for timely interventions, preventing pests from establishing and spreading. Early detection is one of the most significant benefits of pest surveillance. For instance, monitoring for the invasive emerald ash borer (*Agrilus planipennis*) has helped manage its spread and mitigate its impact on ash trees in North America [74, 75]. The timely identification of new or emerging pest threats allows for rapid response measures, including quarantine, eradication, or containment [66, 76]. This proactive approach can prevent the establishment and spread of invasive species, which, once entrenched, can be difficult and expensive to manage. The early detection of the citrus greening disease (Huanglongbing) in Florida facilitated initial control efforts. However, later challenges, such as a prolonged incubation period and regional dispersal, emphasised the importance of robust surveillance systems [77]. Pest surveillance in Australia is essential for the early detection and management of invasive species, minimising their impact on agriculture, biodiversity, and the environment. By providing timely data, surveillance programs help mitigate economic losses and support the protection of Australia’s unique ecosystems [78].

#### Informed decision-making

Surveillance data provide a basis for making informed decisions about pest management strategies. By understanding pest dynamics, authorities can implement targeted control measures that are both effective and environmentally sound [79]. Surveillance data provide critical information that supports decision-making processes in pest management [80]. Stakeholders can develop targeted and efficient pest control strategies by understanding pest distribution, population dynamics, and the environmental factors influencing their spread. This data-driven approach helps allocate resources effectively and prioritise actions based on risk levels [81].

#### Risk assessment and management

Surveillance helps assess the risk posed by potential pest invasions, enabling the development of risk management plans. These plans include contingency measures to address possible outbreaks, thus enhancing biosecurity preparedness [66]. Pest surveillance provides the data necessary for conducting thorough risk assessments by defining the pest status of an area. Surveillance programs can identify which pests pose significant risks and under what circumstances by monitoring pest populations, their spread, and environmental conditions [66]. For instance, the monitoring of the brown marmorated stink bug (*Halyomorpha halys*) in the United States has provided valuable data for risk assessments, helping authorities understand its distribution, host range, and potential to cause economic damage [82].

#### Global collaboration

Pest surveillance requires national and international cooperation to track and manage transboundary pests. Global collaboration is crucial for enhancing plant biosecurity through effective plant pest surveillance. Organisations such as the European and Mediterranean Plant Protection Organization (EPPO) facilitate sharing surveillance data and best practices, strengthening global plant biosecurity [83]. In Australia, the International Plant Sentinel Network (IPSN) is conducting surveillance on five host plants as part of a project funded by the UK Department for Environment, Food and Rural Affairs [84].

#### Pest free areas: achieving and maintaining pest-free areas

Pest-free areas refer to the status of an area that is free from specific pests, achieved through rigorous biosecurity measures and continuous monitoring. Maintaining pest-free areas (PFAs) is crucial for protecting local agriculture, enhancing market access, and reducing pest management’s environmental and economic costs [85]. To prevent the introduction of pests into PFAs, stringent quarantine measures and regulations are necessary. These may include restrictions on the import and movement of plant materials, soil, and other potential carriers of pests. Countries such as Australia have implemented rigorous biosecurity measures to protect their PFAs, with strict controls on the importation of plant products and regular inspections at international borders and ports of entry [86, 87]. As a large, climatically and biologically diverse country with unique flora and fauna, achieving and maintaining pest-free areas in Australia is critical for protecting agricultural production and biodiversity and optimising trade. This is accomplished through rigorous quarantine measures, surveillance, and biosecurity protocols designed to prevent the introduction and spread of pests and ensure compliance with international standards [88, 89]. Collaborative efforts between government agencies, industry stakeholders, and local communities are key to sustaining pest-free status and supporting Australia’s access to global markets [85, 90].

#### Strategies for maintaining pest-free areas

- Strict quarantine regulations and phytosanitary measures prevent the introduction of pests into pest-free areas. These measures include inspecting and treating plants and products before entering new regions [91, 92].
- Continuous monitoring and surveillance programs are essential to ensure that pest-free areas remain pest-free. Advanced monitoring techniques, such as pheromone traps and molecular diagnostics, enhance detection capabilities [93, 94].
- Engaging local communities in biosecurity efforts is vital for maintaining pest-free status. Public awareness campaigns and community-based monitoring programs can help detect and report pest sightings early, facilitating rapid response [95].
- Strong legislative frameworks support the enforcement of biosecurity measures. National and international regulations, such as those set by the IPPC, provide the legal basis for implementing and maintaining pest-free areas [41].

#### Government agencies: policy and regulatory impacts

Government agencies are pivotal in shaping the landscape of plant biosecurity. Their policies and regulatory frameworks can strengthen plant biosecurity or inadvertently create risks.

### Risk creation by government actions

#### Regulatory enforcement

Weak regulatory frameworks can lead to inadequate control over the import and movement of plant materials, increasing the risk of introducing invasive species and pathogens. For instance, insufficient quarantine measures have historically allowed pests like the emerald ash borer (*Agrilus planipennis*) to spread, causing extensive damage to North American forests [74]. Regulatory bodies ensure compliance with biosecurity measures, such as inspecting imports, certifying plant materials, and enforcing quarantine restrictions. This enforcement is vital for preventing the introduction of invasive species that could threaten agricultural productivity and ecosystem health [58]. Regulatory enforcement of plant biosecurity in Australia is governed by the *Biosecurity Act 2015*, which provides a legal framework for preventing, managing, and responding to biosecurity risks [96, 97]. The Department of Agriculture, Fisheries, and Forestry (DAFF) enforces strict import conditions and quarantine measures to protect against the introduction of harmful pests and diseases, such as Khapra beetle (*Trogoderma granarium*) and *Xylella fastidiosa* [87]. Compliance with these regulations is ensured through inspections, audits, and penalties for breaches, which are crucial for maintaining Australia’s pest-free status [98–100]. Collaboration between state and federal governments ensures a coordinated approach to enforcement, supported by surveillance programs and risk assessments to manage potential threats.

#### Funding and collaborations

Lack of funding for research, surveillance, and enforcement of biosecurity measures hamper detecting and responding to pest outbreaks. Budget cuts to agricultural departments can limit the effectiveness of pest management programs, making ecosystems more vulnerable to invasive species and causing an impact on plant biosecurity, which affects food safety and security [101]. Government agencies often collaborate with international organisations and other countries to harmonise biosecurity standards and respond to global threats. Collaborative research is vital in One Health, as plant biosecurity is intrinsically linked to the broader ecosystem and human health. For example, agencies may participate in international agreements such as the IPPC to coordinate global responses to plant biosecurity threats [4]. Through initiatives like the Agricultural Competitiveness White Paper and the Biosecurity Innovation Program, the Australian government provides substantial funding to support biosecurity research, surveillance, and response activities [102, 103]. Collaborative efforts between government agencies, research institutions, industry bodies, and international partners enhance the effectiveness of biosecurity measures, including the development of new technologies and shared knowledge [104]. These partnerships are essential for the early detection of biosecurity threats, rapid response, and ongoing protection of Australia’s agricultural and natural environments [105–107].

#### Policy gaps and inconsistencies

Inconsistent policies across international regions can create biosecurity loopholes. For example, variations in phytosanitary standards between countries can facilitate the cross-border movement of pests. Effective biosecurity requires harmonised policies and standards, as emphasised by the IPPC [41]. Government agencies are responsible for creating policies establishing the legal framework for plant biosecurity. These policies include quarantine regulations, pest surveillance programs, and the enforcement of international standards [91, 108]. Effective policy implementation is crucial for maintaining biosecurity and preventing the cross-border movement of harmful pests and pathogens [59]. The policies and regulations established by government agencies have direct implications for the One Health approach. For instance, stringent biosecurity measures that prevent plant disease outbreaks also reduce the need for chemical interventions, which can harm human and animal health. Conversely, inadequate regulation can lead to the spread of plant pathogens, which may disrupt ecosystems and increase the risk of zoonotic disease transmission [5]. Environmental and plant biosecurity are often overlooked due to the more immediate and visible impacts of zoonotic diseases on human and animal populations [109], while plant threats tend to be perceived as slower-moving and less urgent. Additionally, economic and political pressures often focus on short-term outcomes, neglecting the long-term, systemic risks posed by plant and environmental health vulnerabilities [110, 111].

#### Mitigation and positive contributions

Governments can mitigate risks by implementing and enforcing robust biosecurity regulations. The European Union’s Plant Health Regulation, which aims to prevent the introduction and spread of plant pests within the EU, exemplifies a solid, robust regulatory framework [112]. Government agencies implement various mitigation strategies to prevent and manage plant biosecurity threats. These include quarantine measures, pest surveillance, and the enforcement of strict import and export regulations to control the movement of plants and plant products [113]. Such measures help reduce the risk of introducing invasive species and plant pathogens that can have devastating effects on agriculture and natural ecosystems [58]. Research and development are crucial for advancing pest detection, surveillance, and management technologies. Government-funded research institutions, such as the Commonwealth Scientific and Industrial Research Organisation (CSIRO) and Hort Innovation in Australia and the United States Department of Agriculture (USDA), are critical in developing innovative solutions to plant health challenges [114]. The development of robust regulatory frameworks is a crucial responsibility of government agencies. These frameworks provide the legal foundation for biosecurity measures, ensuring that there are clear guidelines and enforcement mechanisms in place to protect plant biosecurity [115]. Effective regulations also facilitate international trade by ensuring that countries meet phytosanitary standards, thereby preventing the spread of pests and diseases across borders [59].

Governments can enhance global plant biosecurity through international agreements and collaborations. Participation in global initiatives like the IPPC facilitates the sharing of information, resources, and best practices, strengthening collective biosecurity efforts [41]. Government agencies contribute to the broader One Health objectives of safeguarding human and animal health by protecting plant biosecurity. For example, by preventing plant disease outbreaks, agencies reduce the need for chemical pesticides, which can have negative health impacts on humans and animals through contamination of food, water, and soil [5, 116]. Additionally, healthy plants and ecosystems are essential for maintaining biodiversity, which supports resilient agricultural systems and reduces the risk of zoonotic diseases [2] and also align with One Health principles by promoting environmentally friendly and health-conscious farming practices [117].

#### Industry: practices and their implications

The agricultural and trade industries are central to plant biosecurity due to their direct interaction with plant production and movement. While these sectors drive economic growth, certain practices can create risks for plant biosecurity.

### Risk creation by industry actions

#### Agricultural practices

The agricultural industry heavily influences plant biosecurity through its cultivation practices, pest management strategies, and agricultural inputs such as fertilisers and pesticides. Practices like monoculture farming, which involves growing large areas of a single crop, can exacerbate pest problems by creating environments conducive to pest proliferation [8]. The adoption of integrated pest management (IPM) practices, which combine biological, cultural, and chemical controls, can enhance plant biosecurity while aligning with One Health principles by minimising environmental and health risks [118]. Additionally, overreliance on chemical pesticides can lead to resistance in pest populations, making them harder to control [119]. Agricultural practices in Australia, such as large-scale monocropping, the overuse of chemical pesticides, and intensive livestock farming, can inadvertently create biosecurity risks by promoting pest resistance and environmental degradation. These practices can increase the vulnerability of crops and ecosystems to invasive species, pests, and diseases, undermining both plant and animal health [120–122]. Additionally, improper waste management and the movement of agricultural goods can facilitate the spread of pests and pathogens across regions, exacerbating biosecurity challenges [123, 124].

#### Supply chain vulnerabilities

Complex and fragmented supply chains can obscure products’ origins and health status, making tracking and managing pest risks difficult. Lack of transparency and traceability in supply chains can hinder rapid response to pest outbreaks. Industry practices related to supply chain management, including sourcing, handling, and transporting plant materials, are critical points for biosecurity intervention [125, 126]. Poor management practices can lead to the accidental spread of pests and diseases, whereas strict adherence to phytosanitary standards and biosecurity protocols can mitigate these risks [58].

#### National/global trade and movement of goods

The national or global trade of plants and plant products increases the risk of spreading pests and diseases [127]. The movement of infested goods across borders can lead to the establishment of pests in new areas. For example, global trade in ornamental plants has been a significant pathway for spreading invasive pests such as red imported fire ants (*Solenopsis invicta*) [128]. The agricultural industry is a major driver of global trade, and its practices have significant implications for plant biosecurity [63, 129]. Trade policies and agreements must balance economic growth with biosecurity measures to prevent the spread of pests and diseases. Industry compliance with international phytosanitary standards is essential for maintaining trade while protecting plant biosecurity. Non-compliance can lead to trade restrictions, economic losses, and biosecurity breaches with far-reaching consequences [58].

#### Mitigation and positive contributions

Industries can mitigate risks by adopting best practices for biosecurity, such as implementing rigorous hygiene protocols, using certified pest-free planting material, and promoting IPM strategies. Integrated pest management reduces the reliance on chemical controls and encourages sustainable pest management practices [67]. The agricultural industry can invest in technological innovations to improve pest detection and management. Precision agriculture technologies, such as drones and remote sensing, enable more efficient monitoring and management of pest populations [46].

Companies can incorporate biosecurity into their corporate social responsibility initiatives, promoting environmental, sustainability and governance (ESG) for biosecurity and pesticide management [130], and supporting community-based pest management programs [131]. Industry leaders can influence positive change by prioritising plant biosecurity and sustainability in their business models. The industry has a role in raising public awareness about plant biosecurity. Corporate social responsibility (CSR) initiatives can include education campaigns, partnerships with governments and NGOs, and investment in community-based biosecurity programs. By engaging with the public and promoting responsible practices, industries can help foster a culture of biosecurity that supports One Health [20]. For instance, organic farming practices, which emphasise soil health and natural pest control methods, offer a model for integrating plant biosecurity with environmental and public health objectives [132].

#### Socio-political aspects of One Health

The socio-political aspects of One Health and plant biosecurity encompass the complex interplay between government policies, industry practices, community engagement, and global cooperation [133, 134]. The potential loss of global fisheries, forests, and water resources is compounded by the lack of a unified framework to understand complex social-ecological systems (SESs), as scientific disciplines often use differing concepts and languages. Recent research challenges the assumption that only governments can manage resources, showing that in some cases, resource users successfully self-organise to achieve sustainability, while specific government policies can exacerbate resource degradation [135]. Evaluation of the adequacy and organisation of internal and external resources within complex systems, shifting attention from specific disease causes to the underlying goals and structure of the system to improve health outcomes, particularly in agroecosystems [136]. Integrating health and well-being into watershed governance emphasises that well-managed watersheds reduce health hazards and strengthen social-ecological resilience and community empowerment. It introduces the Watershed Governance Prism to guide decision-making and policy development, linking ecosystem, social, and health benefits to foster sustainable, equitable, and resilient water-land systems [137]. In Australia, plant biosecurity falls under state and federal jurisdictions, requiring strong coordination to effectively address pest and disease threats. This multi-level governance structure often leads to challenges in implementing uniform biosecurity measures across regions, as different stakeholders have varied interests and priorities [138]. Political commitment is not just important but crucial, as government funding and legislation directly impact the development of surveillance systems, quarantine protocols, and rapid response mechanisms for plant biosecurity. This support is vital for the success of plant biosecurity measures and preventing the spread of harmful organisms [127, 139].

Additionally, the involvement of local communities, including Indigenous groups, is essential in biosecurity practices, as they often have valuable traditional knowledge and are directly affected by plant health policies. Public awareness campaigns are vital to fostering community participation in biosecurity efforts, ensuring early detection and reporting of pests or diseases [131, 140]. International cooperation also plays a key role, as biosecurity threats can cross borders, necessitating collaboration with other countries to prevent the spread of invasive species through trade and travel [141]. One Health principles add another layer to the socio-political discussion, as they call for integrated approaches that consider human, animal, plant, and environmental health together. Incorporating plant biosecurity into One Health strengthens biosecurity by recognising the interdependence between agricultural productivity, food security, and ecosystem health. This approach encourages more inclusive, cross-sectoral policymaking that balances agricultural needs with environmental conservation [142–144]. However, achieving such collaboration requires overcoming political, institutional, and economic barriers often hindering coordinated action.

## Challenges and opportunities of One Health approach in plant biosecurity

The One Health approach in plant biosecurity faces challenges, particularly in its limited integration across sectors in the broader One Health framework, as plant biosecurity is often excluded from mainstream discussions focused on human and animal health [145]. This exclusion can lead to gaps in biosecurity systems, reducing the effectiveness of measures designed to prevent the spread of plant pests and diseases, which have significant implications for ecosystems and food security [14, 146]. One of the main challenges is the lack of intersectoral coordination, as plant biosecurity involves multiple stakeholders across the agriculture, trade, environment, and health sectors [18, 147]. Another challenge is resource constraints, particularly in low- and middle-income countries (LMICs), where regulatory bodies may lack the capacity to implement effective plant biosecurity measures. However, the One Health approach offers significant opportunities. It promotes a more holistic view of biosecurity by recognising the interconnectedness of plant, animal, and human health, essential for sustainable food systems and ecosystem resilience. For example, incorporating plant biosecurity into One Health frameworks can help mitigate the risk of zoonotic diseases, which may arise from disrupted ecosystems caused by agricultural practices [148, 149]. Another opportunity lies in developing new surveillance systems that monitor the health of plants, animals, and humans together, thus enabling earlier detection of potential threats and more effective interventions [150, 151]. Applying One Health in plant biosecurity can improve cross-border cooperation, especially in regions prone to invasive species and pests, such as Africa and Southeast Asia [129, 152]. Furthermore, enhancing regulatory capacities in LMICs through One Health can strengthen the ability to assess risks like the emergence of antimicrobial resistance threats and implement effective biosecurity measures to combat the risks [153, 154]. While challenges remain regarding coordination and resource allocation, the One Health approach presents a promising avenue for advancing plant biosecurity through integrated health and ecosystem management.

## Conclusion

Plant biosecurity is a critical element of the One Health approach, ensuring that the life and health of plants, humans, animals, and ecosystems are maintained. Plant biosecurity within the One Health framework is significant in maintaining the health of plants, humans, animals, and ecosystems. We can protect plant biosecurity and enhance global resilience through effective pest surveillance, rigorous biosecurity measures, and sustainable pest management practices. Effective pest surveillance and biosecurity measures are crucial for preventing the introduction and spread of harmful plant pathogens, which can affect food security and ecosystem stability. The interconnectedness of plant biosecurity with overall health highlights the importance of integrating plant biosecurity into broader health and environmental policies. Adopting this integrated approach helps build a more resilient and sustainable future where plant health contributes to the planet’s well-being. Ultimately, the interconnectedness of plant biosecurity and One Health emphasises the need for collaborative efforts and interdisciplinary solutions to address complex global challenges. As we continue to face global challenges such as food security, biodiversity loss, and climate change, prioritising plant biosecurity within the One Health framework is essential for a sustainable and healthy future. Urgent improvements are needed in biosecurity policy areas, such as early detection systems, rapid response frameworks, and stricter import regulations to prevent the introduction of invasive pests and diseases. Strengthening international collaborations, particularly in data sharing and coordinated pest surveillance, will enhance global plant biosecurity within the One Health framework. The suggested strategies emphasise stronger collaboration between government and industry within a One Health framework, leading to improved regulatory policies, enhanced pest surveillance, and more effective risk mitigation measures, ultimately safeguarding plant health, biodiversity, and agricultural sustainability.

### Limitations

This study is limited by the availability and consistency of data on plant biosecurity measures across different regions, which may affect the generalisability of findings. Additionally, the study primarily focuses on government and industry roles, potentially overlooking the contributions of other stakeholders such as local communities. A key limitation is the limited comparison with international experiences beyond Australia, which could provide broader insights into biosecurity and One Health strategies. Furthermore, while the One Health framework is emphasised, the complex interactions between plant, animal, and human health require further interdisciplinary research beyond the scope of this study. Furthermore, this is a narrative review, and the data search approaches were not a systematic way as we searched PubMed, Scopus, and Web of Science using the keywords: (“Plant biosecurity” OR “One Health” OR Governance OR Industry OR Surveillance OR Policy), without any timeframe of publication for this review.

## Data Availability

No datasets were generated or analysed during the current study.
